# Prostate cancer immunotherapy: Improving clinical outcomes with a multi-pronged approach

**DOI:** 10.1016/j.xcrm.2023.101199

**Published:** 2023-09-21

**Authors:** Dhivya Sridaran, Elliot Bradshaw, Carl DeSelm, Russell Pachynski, Kiran Mahajan, Nupam P. Mahajan

**Affiliations:** 1 Division of Urologic Surgery, Department of Surgery, Washington University in St Louis, Cancer Research Building, 660 S. Euclid Avenue, St Louis, MO 63110, USA; 2 Bursky Center for Human Immunology and Immunotherapy Programs (CHiiPs), Washington University in St Louis, Cancer Research Building, 660 S. Euclid Avenue, St Louis, MO 63110, USA; 3 Department of Radiation Oncology, Washington University in St Louis, Cancer Research Building, 660 S. Euclid Avenue, St Louis, MO 63110, USA; 4 Division of Oncology, Department of Medicine, Washington University in St Louis, Cancer Research Building, 660 S. Euclid Avenue, St Louis, MO 63110, USA; 5 Siteman Cancer Center, Washington University in St Louis, Cancer Research Building, 660 S. Euclid Avenue, St Louis, MO 63110, USA

**Keywords:** prostate cancer, CAR T cell therapy, vaccine, immune checkpoint blockade, ICB, androgen receptor, AR, castration-resistant prostate cancer, CRPC, small-molecule inhibitors, cabozantinib, ibrutinib, CEP-11981, (*R*)-9b, pexidartinib, SGI-9481, TKI, BiTE, CTC

## Abstract

Cancer immunotherapy has gained traction in recent years owing to remarkable tumor clearance in some patients. Despite the notable success of immune checkpoint blockade (ICB) in multiple malignancies, engagement of the immune system for targeted prostate cancer (PCa) therapy is still in its infancy. Multiple factors contribute to limited response, including the heterogeneity of PCa, the cold tumor microenvironment, and a low number of neoantigens. Significant effort is being invested in improving immune-based PCa therapies. This review is a summary of the status of immunotherapy in treating PCa, with a discussion of multiple immune modalities, including vaccines, adoptively transferred T cells, and bispecific T cell engagers, some of which are undergoing clinical trials. In addition, this review also focuses on emerging mechanism-based small-molecule tyrosine kinase inhibitors with immune modulatory properties that, either as single agents or in combination with other immunotherapies, have the potential to improve clinical outcomes.

## Graphical abstract

**Figure:**
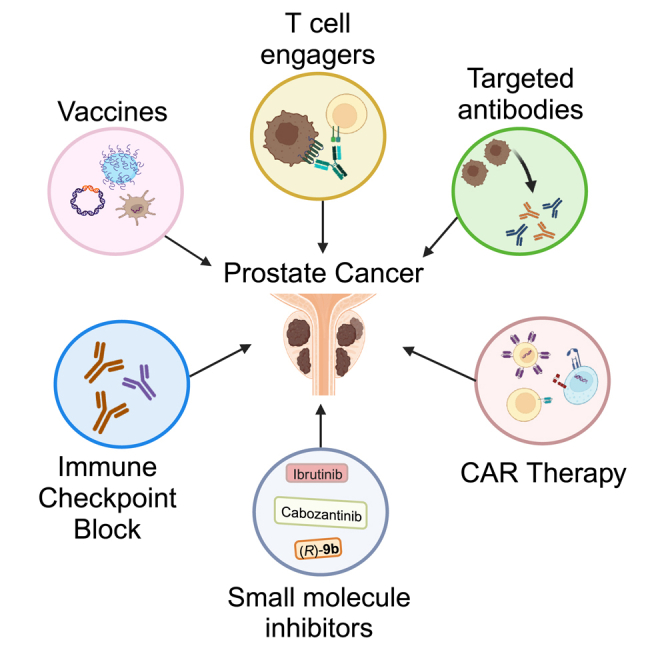

## Introduction

Prostate cancer (PCa) is the second most common cancer among men, consisting of 14% of new cancer cases identified in 2022.^1^ At the molecular level, genomic, epigenomic, transcriptional, and post-translational changes contribute to prostate carcinogenesis to drive tumor heterogeneity.^2^^,^^3^^,^^4^^,^^5^ A large majority of PCas are characterized and driven by abnormalities in androgen and androgen receptor (AR) signaling,^5^^,^^6^ which has resulted in widespread usage of androgen deprivation therapy (ADT). However, a majority of ADT-treated patients develop resistance and advance to an aggressive stage: castration-resistant PCa (CRPC) or metastatic CRPC (mCRPC).^3^^,^^5^ PCa tumors start as AR dependent, but grow independent of testicular androgen, which prompted the development of the AR antagonist enzalutamide, an inhibitor of AR nuclear translocation,^7^ and abiraterone, which inhibits *de novo* androgen synthesis in tumors.^8^ Although highly effective initially, most patients develop resistance, with few therapeutic options available.

Cancer immunotherapy primarily relies on the revival of the compromised host immune system,^9^ which can be amplified through synthetic immunity (e.g., chimeric antigen receptor T cells [CAR Ts], bispecific antibodies [BiTEs]). Immunotherapy has superior efficacy over pharmacological cancer therapy due to its precise targeting and persistence over time, as seen in multiple cancers, such as melanoma, lung, and kidney cancers and leukemia.^10^^,^^11^ Immunoediting and selective pressure on PCa cells resulting in the outgrowth of less immunogenic, apoptosis-resistant neoplastic prostate cells, have prompted efforts to identify and evaluate PCa immunotherapy in combinatorial settings. Here, we discuss the latest advances in PCa immunotherapy and their clinical efficacy and shed light on plausible markers and targets complementing our current understanding of effective immune stratification and treatment.

## Immune checkpoint inhibitors

Targeting the co-inhibitory pathways of T cells, termed T cell checkpoints,^12^ elicits an antitumor response by shifting the balance from T cell inhibition by co-inhibitory molecules, like LAG-3, PD-1, TIM-3, and CTLA-4, to enhanced pro-inflammatory conditions that no longer support tumor growth.^13^ Immunotherapy for solid tumors predominantly uses inhibitors of programmed cell death-1 (PD-1)/programmed cell death ligand-1 (PD-L1) and cytotoxic T lymphocyte antigen-4 (CTLA-4) and is referred to as immune checkpoint blockade (ICB).^14^^,^^15^ The monoclonal antibodies commonly used in ICB include nivolumab, which blocks PD-1, and ipilimumab, which targets CTLA-4.^11^ However, a clinical trial of nivolumab in mCRPC patients showed no significant objective response rates.^16^ Subsequently, clinical studies of pembrolizumab, another anti-PD-1 monoclonal antibody, in mCRPC patients were ineffective, indicating that the application of PD-1 inhibitors as single agents and use of PD-L1 expression as a marker to establish sensitivity to PD-1 blockade therapy are insufficient to treat advanced PCa.^17^ Both a phase II clinical trial of ipilimumab in synergy with radiotherapy in mCRPC patients and a phase III trial in patients who previously received chemotherapy showed no differences in objective response rate between placebo and ipilimumab.^18^ mCRPC patients with a high intratumoral CD8^+^ T cell density had favorable responses to CTLA-4-targeting antibodies,^19^ suggesting that enhanced CD8^+^ activation may show clinical benefits. Intriguingly, a long-term follow-up study of mCRPC patients treated with ipilimumab showed favorable overall survival (OS) rates at 3, 4, and 5 years that were approximately two to three times higher than those of patients on the control arm, suggesting long-term benefit of immunotherapy in these patients.^18^^,^^20^

The limited activity of single-agent checkpoint inhibition in patients suggests a need for combination therapy. To expand immunotherapy benefits, dual checkpoint blockade has been evaluated in the randomized, controlled CheckMate 650 clinical trial. This trial evaluated two dosing regimens of ipilimumab/nivolumab, compared with single-agent ipilimumab and the standard-of-care chemotherapy cabazitaxel. Treatment with ipilimumab (3 mg/kg) and nivolumab (1 mg/kg) showed the highest overall response rate (19.5% vs. 12.2%) and complete response rate (4.9% vs. 0%) compared with cabazitaxel,^21^ with a prolonged duration of radiographic response (6.5 months vs. non-responders [NR]), providing evidence of meaningful clinical activity in these patients. With more ongoing clinical trials in different combinatorial settings, ICB is expected to exhibit improved OS in PCa patients.

## Vaccines

Most cancer vaccines consist of DNA/RNA/peptides that impart antigen-specific immune responses through antigen presentation and activation of naive T cells. Commonly targeted antigens in PCa vaccine development include prostatic acid phosphatase (PAP), prostate-specific antigen (PSA), prostate-specific membrane antigen (PSMA), prostate stem cell antigen (PSCA), and six-transmembrane epithelial antigen of the prostate-1 (STEAP1), due to their overexpression and enrichment in tumors compared with the normal prostate.

### DNA-based cancer vaccines

Circular DNA plasmids that encode tumor antigens are used as vaccines and are taken up robustly by antigen-presenting cells (APCs) to activate adaptive immunity through major histocompatibility complex (MHC) class I and II-restricted antigen presentation to CD8^+^ and CD4^+^ T cells.^22^ These vectors also produce an adjuvant effect^23^ via the TLR9^24^ or cyclic GMP-AMP synthase (cGAS)-STING pathway^25^ owing to their unmethylated CpG DNA. pTVG-HP is a DNA vaccine undergoing clinical trial that elicits PAP-targeting CD4^+^ and CD8^+^ T cell responses in PCa patients.^26^^,^^27^ Another DNA-based vaccine, pTVG-AR, targets the AR ligand-binding domain (AR LBD). A randomized phase II trial evaluating these two vaccines in combination with pembrolizumab in mCRPC patients is currently underway (Table 1). A study in metastatic castration-sensitive PCa patients receiving ADT showed that pTVG-AR imparted Th1-type antitumor immunity in 47% of patients, promising significantly prolonged progression-free survival.^28^

**Table 1:** Clinical trials of immunotherapy and its combinations for prostate cancer

Drug/cells	Nature	Therapy and mechanism of action	Disease	Trial phase	Recruiting status	Trial identifier
Nivolumab	anti-PD1 antibody	prevents PD-L1 and PD-L2 from inhibiting the action of T cells by binding to PD-1	metastatic PCa	II	completed	NCT03651271
Ipilimumab	anti-CTLA-4 antibody	humanized monoclonal antibody blocking cytotoxic T lymphocyte antigen-4 (CTLA-4)	metastatic PCa	III	completed	NCT01057810
Sipuleucel-T (Provenge)	DC vaccine with PA2024 (PAP) and granulocyte-macrophage colony-stimulating factor fusion	autologous cellular immune therapy	metastatic PCa after failing hormone therapy	III	completed	NCT00065442
pTVG-HP vaccine with or without pTVG-AR DNA vaccine and pembrolizumab	PAP-targeted DNA vaccine	concurrent targeting of PAP and blockade of PD-1 using pembrolizumab (two-vaccine strategy) to improve tumor-directed CD8^+^ T cells	metastatic CRPC	II	recruiting	NCT04090528
PROSTVAC (PSA-TRICOM)	recombinant viral vaccine targeting PSA	targeting PSA and a triad of co-stimulatory molecules (TRICOM), LFA-3, B7.1, and ICAM-1, for greater T cell activation; utilizes antigen spreading phenomenon	localized PCa	II	completed	NCT02326805
PROSTVAC and docetaxel	combination of recombinant viral vaccine targeting PSA with antineoplastic agent	combination of two different live poxvirus-based vectors: PROSTVAC-V, a recombinant vaccinia virus, and PROSTVAC-F, a recombinant fowlpox virus with docetaxel chemotherapy	metastatic castration-sensitive PCa	II	active	NCT02649855
PROSTVAC, CV301, and MSB0011359C (M7824)	combination of recombinant viral vaccine targeting PSA, bifunctional fusion protein, and recombinant Avipoxvirus	combination of PROSTVAC-V, PROSTVAC-F, monoclonal antibody with bifunctional fusion protein comprising IgG1 anti-PD-L1 and TGF-βRII, and recombinant Avipoxvirus encoding two tumor-associated antigens, CEA and MUC-1	biochemically recurrent PCa	II	recruiting	NCT03315871
ETBX-071, ETBX-061, and ETBX-051	combination of recombinant adenovirus vaccines	combination of recombinant Ad5 PSA/MUC1/Brachyury vaccines that induce T cell-mediated immune responses against at least one of the three tumor-associated antigens	CRPC	I	completed	NCT03481816
NY-ESO-1 protein	peptide vaccine	NY-ESO-1 protein is combined with CpG oligonucleotide adjuvant CpG 7909 to activate immunological response	advanced PCa	I	completed	NCT00292045
UV1	synthetic peptide vaccine	human telomerase fragment, UV1 peptide in combination with GM-CSF to immunologically target cancer cells	hormone-sensitive metastatic PCa	I/II	active	NCT01784913
PSCA-CAR T cells	chimeric antigen receptor T cells	autologous anti-PSCA-CAR-4-1BB/TCRζ-CD19t-expressing T lymphocytes in combination with chemotherapy drugs cyclophosphamide, fludarabine, and fludarabine phosphate	metastatic CRPC	I	recruiting	NCT03873805
PD1-PSMA-CAR T cells	chimeric antigen receptor T cells	non-viral programmed cell death protein-1 (PD-1) integrated anti-prostate-specific-membrane-antigen (PSMA) CAR T cell immunotherapy	CRPC	I	recruiting	NCT04768608
PSMA-targeted CAR T cells	chimeric antigen receptor T cells	CAR T cell immunotherapy with chimeric antigen receptor targeting PSMA	CRPC	I	recruiting	NCT05354375
TABP EIC	chimeric antigen receptor natural killer (NK) cells	experimental interventional therapy with combination of anti-PSMA-targeted CAR NK cell immunotherapy with chemotherapy drugs cyclophosphamide and fludarabine	metastatic CRPC	I	recruiting	NCT03692663
CC-1	bispecific antibody	bispecific antibody (bsAb) with PSMA × CD3 specificity that binds to PSMA on cancer cells and tumor vessels allowing dual anticancer action	CRPC	I	recruiting	NCT04104607
AMG 509	bispecific antibody	STEAP1 × CD3 XmAb 2 + 1 bispecific antibody that simultaneously binds to STEAP1 on tumor cells and CD3 complex on T cells causing T cell-mediated lysis of STEAP1-expressing cells in combination with chemotherapy drugs abiraterone or enzalutamide or docetaxel	metastatic CRPC	I	recruiting	NCT04221542
Tarlatamab (AMG 757)	bispecific antibody	bispecific T cell-engager molecule targeting inhibitory notch ligand delta-like ligand 3	neuroendocrine PCa	I	recruiting	NCT04702737
JNJ-78278343	bispecific antibody	humanized immunoglobulin (Ig) G1-based bispecific antibody that binds to the CD3 receptor complex of T cells and KLK2 on target tumor cells causing T cell-mediated lysis of the KLK2 bearing tumor cells	advanced PCa	I	recruiting	NCT04898634
LAVA-1207	bispecific antibody	humanized Fc-containing bispecific antibody that engages PSMA and the Vδ2-T cell receptor chain to mediate potent targeting PSMA-expressing cells	therapy refractory metastatic CRPC	I/II	recruiting	NCT05369000
CCW702	bispecific antibody	antibody has a small-molecule imaging agent ligand (DUPA) with specificity for PSMA conjugated to an anti-CD3 antibody through an unnatural amino acid	metastatic CRPC	I	active	NCT04077021
Acapatamab	bispecific antibody	half-life-extended anti-PSMA × anti-CD3 bispecific T cell engager in combination with pembrolizumab (PD-1 inhibitor) or etanercept (TNF-α inhibitor) or cytochrome P450 (CYP) cocktail	metastatic CRPC	I	active	NCT03792841
Cabozantinib (XL184)	small-molecule kinase inhibitor	multi-kinase inhibition targeting tumor cell growth	metastatic CRPC	III	recruiting	NCT04446117
Cabozantinib (XL184) and atezolizumab	small-molecule kinase and immune checkpoint inhibitors	combination of multi-kinase inhibitor cabozantinib, targeting cell growth, and immune checkpoint inhibitor atezolizumab, binding to PD-L1 in cancer cells	metastatic CRPC	II	recruiting	NCT05168618
Ibrutinib	small-molecule kinase inhibitor	neoadjuvant therapy targeting Bruton’s tyrosine kinase modulating B cell signaling and MMP-2 and MMP-9 in cancer cells	localized PCa	II	recruiting	NCT02643667

### RNA vaccines

In contrast to DNA vaccines, mRNA vaccines have several inherent advantages, as they are well tolerated, lack oncogenic risk,^29^ are highly specific and immunogenic^30^ regardless of MHC haplotype, and do not require translocation into the nucleus for activation. CV9103 is a self-adjuvant mRNA encoding PSA, PSMA, PSCA, and STEAP1 and, in an early clinical trial, induced a significant immune response improving OS.^31^ Further development of mRNA vaccines encoding specific highly immunogenic PCa antigens may show promise for novel therapeutic intervention.

### Other nucleic acid vaccines

Spherical nucleic acids are a class of nanostructures containing CpG oligonucleotides as an adjuvant and the prostate tumor antigens PSA, PSMA, and PAP, which have improved cross-priming of antitumor CD8^+^ T cells in PCa models.^32^ A phase I/II trial of a DNA fusion vaccine that encodes the fragment C domain of tetanus toxin linked to an HLA-A2-binding epitope from PSMA showed specific CD4^+^ and CD8^+^ T cell-mediated antitumor responses in CRPC patients.^33^

### Adenoviral vaccines

The use of adenoviruses for cancer vaccines has been favored due to their episomal genomic nature, reducing chances of insertional mutagenesis.^34^ A phase I clinical trial of the TriAdeno vaccine, consisting of Ad5 vectors encoding the antigens CEA, MUC-1, and Brachyury, showed significant activation of CD4^+^ and/or CD8^+^ T cell responses in mCRPC patients without antigenic competition.^35^ Currently, an Ad5-PSA vaccine is in a phase II trial (NCT00583024) and is expected to improve anti-PSA T cell responses in hormone-refractory cases and inhibit recurrent disease in PCa patients.

### Dendritic cell vaccines

Sipuleucel-T is a dendritic cell (DC) vaccine consisting of PA2024 (PAP) with granulocyte-macrophage colony-stimulating factor fusion and is the only approved active cellular immunotherapy for mCRPC.^36^ Sipuleucel-T administration to patients before prostatectomy induced both T and B cell-associated sustained immune responses,^37^ reduced PSA levels, and improved OS.^36^ Studies with different combinations and clinical settings are underway, such as combination with approved mCRPC drugs, antibodies, or radiation therapy. A recent randomized phase II trial evaluated the combination of Sipuleucel-T with or without the addition of the homeostatic cytokine interleukin-7 (IL-7) and showed significant expansion of lymphocyte populations and increased immune responses with a decrease in PSA in patients receiving the combination compared with Sipuleucel-T alone,^38^ suggesting combinatorial approaches hold promise for improving clinical efficacy.

### Fusion proteins as vaccines

**Figure 1:**
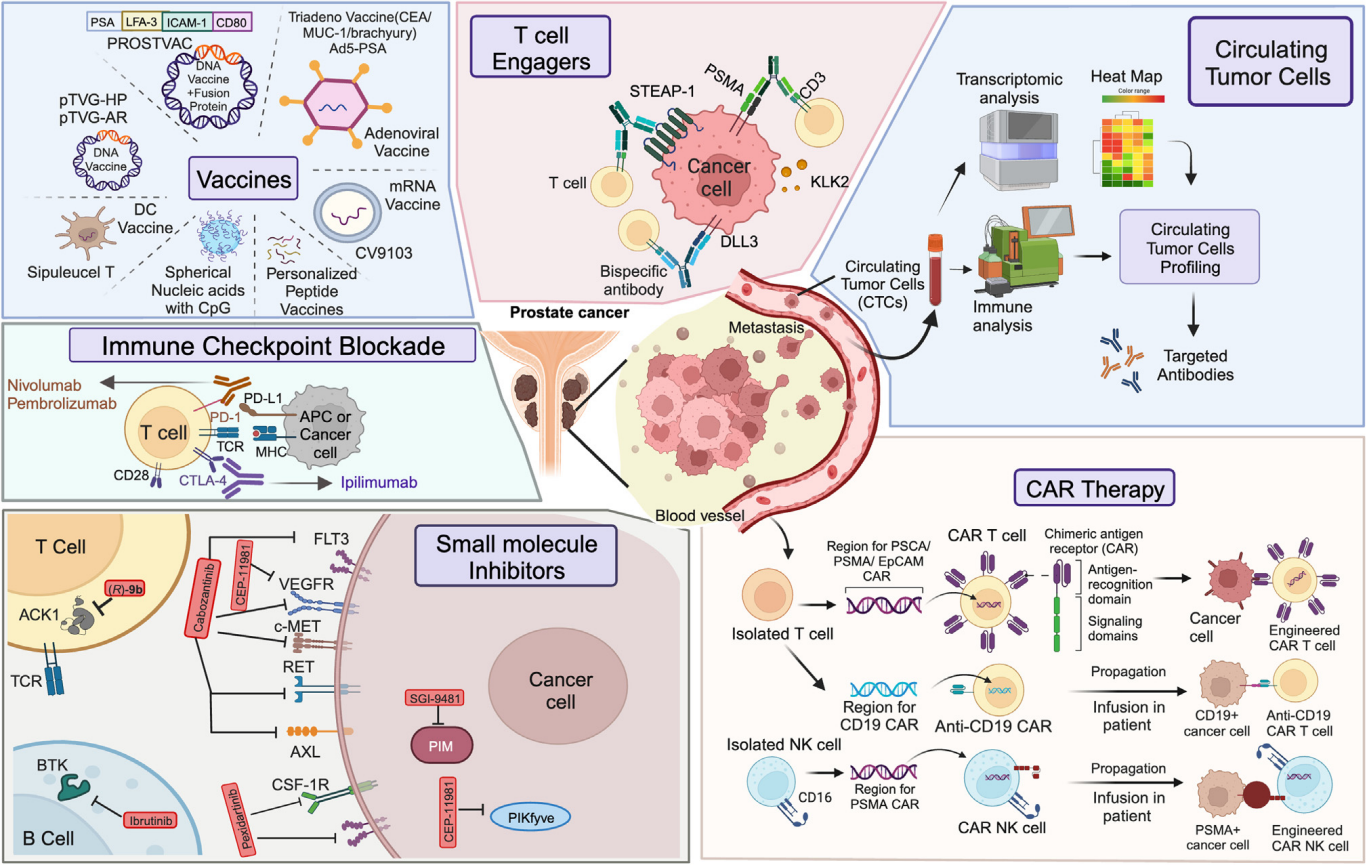
Current and emerging immunotherapeutic options to treat prostate cancer Shown are the existing and emerging strategies for PCa immunotherapy. The classic immune checkpoint blockade (ICB) therapy uses monoclonal antibodies against PD-1/PD-L1/CTLA-4. ICB in combination with other therapies is currently being tested to maximize efficacy. Several DNA/RNA peptide vaccines have shown promise in inhibiting PCa growth. Generation and testing of novel fusion proteins and nucleic acid formulations are underway. T cell engagers and bispecific antibodies (BiTEs) that create cancer-destroying contact between immune cells and cancer cells is another proficient immunotherapy strategy gaining prominence. Profiling of circulating T cells (CTCs) to screen treatment-induced antigenic alterations and novel marker detection is emerging as an effective strategy for personalized therapeutic intervention ensuring generation of targeted antibodies with improved clinical efficiency. Multiple immune cell types bearing chimeric antigen receptors (CARs) are being engineered and tested for therapeutic efficacy against PCa. Targeting tyrosine kinases of molecular significance contributing to immunosuppression using small-molecule inhibitors and their combination with other immunotherapies also hold promise in improving existing PCa treatment regimens. Image was generated using BioRender.com.

PROSTVAC (PSA-TRICOM) is a recombinant viral vaccine that targets PSA and incorporates B7.1, ICAM-1, and LFA-3 co-stimulatory molecules (Figure 1). Clinical trials in castration-sensitive and CRPC patients show that PROSTVAC efficiently activates cytotoxic T lymphocytes upon antigen presentation.^39^ Other combinations include neoadjuvant/adjuvant and immune checkpoint inhibitors^40^ with docetaxel, ipilimumab (NCT02506114), and recombinant Avipoxvirus vaccine CV301, a mucin-1 targeting viral vaccine (Table 1). A DC-based vaccine with a fusion protein containing a secretin-penetratin (SecPen) peptide, New York esophageal squamous cell carcinoma-1 (NY-ESO-1), and ubiquitin was constructed to enable targeted antigen presentation by DCs and elicited strong T cell responses in mice against murine MC38 colon carcinoma cells.^41^ Similar cellular vaccines with fusion proteins hold great promise to efficiently curb PCa progression.

### Tumor-associated proteins and neoantigens

Cancer testis antigens are a vast antigen group of over 70 gene families that are highly expressed in tumors and elicit significant immune responses in cancer patients.^42^ NY-ESO-1 or cancer-testis antigen 1B (CTAG1B) is expressed by ∼25% of PCa cells on their surface. Transglutaminase 4 (TGM4) expression is primarily confined to the luminal epithelia of prostate tumors, and TGM4-pulsed monocyte-derived DCs (moDCs) expanded CD8^+^ and CD4^+^ T cells, thus making TGM4 a potential vaccine candidate to treat PCa.^43^ Receptor activator of nuclear factor κB ligand (RANKL) is activated by cytokines during PCa metastasis to bone and causes bone resorption. Recent studies have shown that RANKL-immunized mice showed reduced metastasis of PCa cells and inhibited the WNT-β-catenin signaling that is predominantly activated in aggressive cancers.^44^

A limited number of trials evaluating neoantigen vaccine usage have been performed to date. The first trial of neoantigen vaccines in metastatic PCa was recently reported^45^; this single-arm trial combined dual ICB (ipilimumab and nivolumab) with a DNA-based vaccine composed of ∼20 neoantigens in patients with metastatic hormone-sensitive PCa (mHSPC). Overall, the combination was well tolerated, with only two related grade 3 events (colitis) and no grade 4 toxicity. Clinical response evaluations and immune-correlative studies are ongoing.

In addition, telomerase (hTERT) imparts stemness to cancer cells and is a marker for poor prognosis.^46^ A peptide vaccine with a 16-amino-acid hTERT fragment (UV1) in a phase I/II trial produced moderate CD4 (Th1) T cell responses.^47^ Cell-division-associated 1 (CDCA1) is an oncogene overexpressed in PCa; subcutaneous injections of CDCA1 peptide vaccine in docetaxel treatment-failed CRPC patients showed that the vaccine was well tolerated and significantly increased OS among patients in whom the vaccine strongly induced cytotoxic T lymphocyte activity.^48^

### Personalized vaccines

The concept of personalized peptide vaccination (PPV) involves the screening of appropriate tumor antigens and selection of suitable peptides to be used for patient vaccination. In mCRPC patients, PPV decreased PSA levels and produced cellular and humoral immune activation.^49^ A phase II trial of PPV in combination with low-dose cyclophosphamide in mCRPC showed some decline in PSA levels among a small number of patients in both arms, although no difference on OS, progression-free survival (PFS), or antigen-specific T cell activity was observed.^50^ In addition, multi-peptide vaccines are also being tested, e.g., KRM-20, a 20-peptide vaccine combined with dexamethasone and docetaxel, increased numbers of peptide-specific cytotoxic (IFN-γ-positive) T cells and antibodies, but did not affect PFS or OS.^51^ The conventional approach to selecting peptides for PPV warrants the primary screening of pre-vaccination peptides from patients to assess their T cell-priming efficiency while avoiding adverse reactions. Moreover, selection of multiple epitopes for PPV could also reduce the probability of antigen-negative clone generation or tumor escape, thus making multi-peptide vaccination more effective.

Recent advances in vaccine development indicate that vaccines can improve the existing PCa treatment repertoire. However, studies delineating tumor-intrinsic and -extrinsic resistance mechanisms are needed to improve their immunogenicity and eliminate misguidance of vaccines attributed to the genetic complexity of tumors. Alterations to bypass immune tolerance, such as development of chimeric vaccines termed “xenoantigens” encoding non-autoantigens, will improve efficacy in clinical settings. Finally, stratification for nascent tumor recognition with improved delivery systems could help produce ideal cancer vaccines to treat cold tumors like PCa.

## CAR T cell therapy

CAR T cells are equipped with tumor-specific antigen receptors on their surface that facilitate targeted tumor cell killing.^52^ Being human leukocyte antigen (HLA) independent, CAR T cells recognize intact antigens and have a persistent effect by becoming memory T cells.^53^^,^^54^ The tailored CAR consists of an extracellular single-chain fragment variable (scFv) domain for tumor associated antigen (TAA) recognition; a linker region; a transmembrane region derived from T cells comprising CD8, CD28, or IgG; and an intracellular domain composed of a signaling component or co-stimulatory molecule (Figure 1). CAR T co-stimulatory domain composition has evolved over several iterations of studies to incorporate domains such as CD28, 4-1BB, ICOS, or OX40^55^; however, CD28 or 4-1BB are still most commonly used. The efficacy of CAR T therapy is influenced by the selection of the ideal tumor antigen and T cell expansion, differentiation, and persistence in the patient’s system.^56^

### PSMA CAR T therapy

The prostate is an organ of minimal mutational burden; therefore, the widely studied PCa antigens—PSCA, PSMA, and EpCAM—have been tested for application in CAR T therapy. Anti-PSMA CAR T cell therapy was developed based on previous monoclonal antibody studies^57^^,^^58^ and has progressed to clinical trials. An armored PSMA CAR, expressing a dominant-negative receptor for the immunosuppressive cytokine TGF-β, is in a phase I clinical trial of 13 patients across four dose levels and has reported efficient T cell expansion and one patient with a >98% reduction in PSA, plus three more with partial responses of >30% PSA reduction.^59^ Another phase I clinical trial targeting PSMA in nine patients reported three patients with a PSA decrease of >50% and concordant improvement in PSMA positron emission tomography (PET) imaging. One patient in this study experienced a grade 5 event related to hepatic failure and macrophage-activation syndrome potentially linked to the drug (NCT04249947). A PCa xenograft study using another PSMA-targeting second-generation CAR T therapy combined with low doses of docetaxel (an antimicrotubule chemotherapeutic agent) was applied focally and eliminated tumor cells in mice.^60^ Another ongoing clinical trial of anti-PSMA CAR T therapy aims to be combined with PD-1 blockade in treating mCRPC (NCT04768608), opening new treatment paradigms for CAR T cell therapy as an alternate treatment option for PCa (Table 1).

### PSCA CAR T therapy

Pre-clinical studies of anti-PSCA CAR T cells with CD28 or 4-1BB co-stimulatory molecules showed that CAR T cells with CD28 had better efficacy compared with CD137 or 4-1BB against mouse xenograft tumors.^61^

### EpCAM CAR T therapy

EpCAM is a type I transmembrane glycoprotein overexpressed in most PCa tumors and metastases,^62^ and EpCAM-targeting CAR T cells have proceeded to a clinical trial that is ongoing (NCT03013712).

### γδ T cells

The γδ T cell receptor (TCR) recognizes phosphoantigens independent of HLA molecules, thereby targeting cancer cells of various histotypes, and induces antibody-dependent cell-mediated cytotoxicity (ADCC) and co-stimulation of natural killer (NK) cells. Vγ9Vδ2 T cells isolated from human peripheral blood showed cytotoxicity against prostate and other cancer cells after *in vitro* expansion with zoledronic acid and IL-2.^63^ Interest has developed in harnessing these unique γδ T cell features for next-generation adoptive cell immunotherapies and antibody-based γδ T cell engagers. Several clinical trials are now underway to evaluate the safety and efficacy of these cells.

### NK CAR therapy

Current limitations of CAR T therapy have prompted studies of other immune cells harboring CARs for improved antitumor efficacy. Modified NK cells, anti-PSMA/NK92/CAR cells, showed significant PSMA-specific recognition and antitumor activity.^64^ Alternatively, T cells co-expressing NKG2D (NK activating receptor) and IL-7 showed improved persistence and comparatively minimal exhaustion in a xenograft system.^65^

Despite the profound clinical success of CAR T against specific malignancies, off-target effects such as uncontrolled pro-inflammatory responses termed cytokine release syndrome, antigen escape, off-tumor/on-target effects, and poor trafficking/tumor infiltration greatly challenge its potency.^66^ With sustained efforts in delineating CAR T biology, particularly to improve CAR T persistence and overcome limitations such as tonic signaling by genome editing, the minimal efficacy of immunotherapy to treat PCa can be potentially addressed. The growing field of CAR T cells in PCa therapy also warrants focus on the optimal timing of administration, pre-conditioning regimens, bridging regimens, and combinations with other therapies to achieve success in overcoming stromal barriers and other challenges in advanced metastatic scenarios.

## T cell engagers: Bispecific antibodies

BiTEs are a class of bispecific antibodies that bind both tumor antigen and CD3 on T cells, bringing T cells in close proximity to cancer cells (Figure 1), causing tumor cell killing through TCR engagement, followed by phagocytosis and endosome-mediated apoptosis of cancer cells.^67^ The BiTE-induced T cell-tumor cell immunological synapse ensures reactivation of T cells by promoting T cell proliferation without co-stimulatory molecules^68^ and is MHC independent,^69^ which overall ensures killing of immune-evasive cancer cells and overcomes facultative antigen camouflaging.

The first generation of PCa BiTEs is pasotuxizumab (AMG 212), which targets PSMA and CD3 and has shown promising results in pre-clinical mCRPC settings.^70^ However, its short half-life and response failure in a ^177^Lu-PSMA-617-treated patient^71^ prompted a half-life improved version named acapatamab (AMG 160),^72^ which is currently in clinical trials as a single agent as well as in combination with abiraterone and enzalutamide in mCRPC patients non-responsive to taxane and hormonal therapy (NCT03792841). CC-1 is another PSMA-CD3-targeting antibody in clinical trials that aims for better efficacy upon pre-emptive IL-6R inhibition in CRPC patients.^73^ Similarly, CCW702 is a PSMA-CD3 BiTE comprising an imaging ligand (DUPA), and its efficacy is being studied in mCRPC patients (NCT04077021).^74^ LAVA-1207 is another PSMA BiTE under clinical trial to test tumor-inhibiting potential by activation of Vγ9Vδ2 T cells conditionally upon PSMA cross-linking (NCT05369000). Initial data from the trial have shown a favorable safety profile without high-grade (>2) cytokine release syndrome, and dose escalation is expected to show effective disease regression in therapy-refractory mCRPCs.

Human kallikrein 2 (hK2 or KLK2) is a member of the glandular kallikrein serine proteases family (similar to PSA/KLK3)^75^ and is the tumor cell target of JNJ-78278343, a T cell-targeting IgG1-based BiTE. Clinical trials of the KLK2-BiTE to treat mCRPC patients are ongoing (NCT04898634). Similarly, AMG-509, another BiTE targeting the PCa cell-surface antigen STEAP1, has shown significant efficacy against cancer cells in pre-clinical xenograft models and is currently being evaluated for treatment efficacy in patients (NCT04221542) (Table 1). Apart from clinical trials focused on treatment of aggressive mCRPC, immune targeting approaches are also underway for treatment of neuroendocrine PCa (NEPC), which has reduced or no AR expression. NEPCs present with poor prognosis, and the current standard-of-care treatment options are suboptimal. Tarlatamab (AMG 757) is a half-life-extended BiTE that targets delta-like ligand 3 (DLL3) on NEPC cells^76^ and is currently undergoing clinical evaluation (NCT04702737). Among the multiple tumor antigens targeted for immunogenic therapeutic applications is Glypican-1 (GPC-1), an overexpressed member of heparan sulfate proteoglycan in PCa. A GPC-1-targeted BiTE induced release of inflammatory cytokines and reactivated T cell cytolytic activity against PCa cell lines.^77^

Overall, T cell engagers show promise as an additional method to reactivate the host adaptive immune system, and with continued research, novel PCa markers could be identified to fill the void of knowledge for selection of optimal antigens with high immunogenicity. Extensive evaluation of neoantigens might overcome existing concerns about the safety profile of BiTEs, including immune effector cell-associated neurotoxicity syndrome in patients treated with T cell-engaging therapies.^78^ Identification of new markers along with strategies to tune and expand the circulatory life of BiTEs could potentially improve their clinical efficacy.

## Small-molecule kinase inhibitors

Tyrosine kinase inhibitors (TKIs) are grouped into seven classes depending on their target-binding mode and mechanism of action.^79^ Rapid resistance to ADT and progression of CRPC^80^ have led to renewed interest in small-molecule TKIs that inhibit key oncogenic pathways in cells, producing mixed outcomes. Prominent target kinases for PCa therapy include vascular endothelial growth factor receptor (VEGFR), breakpoint cluster region-Abelson tyrosine kinase (BCR-ABL), phosphoinositide 3-kinase (PI3K), colony-stimulating factor-1 receptor (CSF1R), and tyrosine kinase non-receptor 2 (TNK2, also known as ACK1). The outcomes of monotherapy or circumstantial signs of activity upon clinical application of these TKIs have shown that, in addition to targeting various tumor histotypes,^81^ they elicit antitumor immunity by curbing the compensatory pathways that cause therapy resistance (Figure 1).

### XL-184 (cabozantinib)

Cabozantinib is a multi-kinase inhibitor mainly targeting VEGFR2, hepatocyte growth factor receptor (c-MET), rearranged during transfection (RET), stem cell factor receptor (KIT), AXL, and FMS-related tyrosine kinase (FLT3).^82^ Cabozantinib treatment increased the secretion of neutrophil chemotactic factors in PTEN/p53-deficient murine PCa leading to tumor clearance through neutrophil-mediated innate immune response.^83^ It also exerted immunogenic stress in PCa, causing a unique mode of apoptosis defined by release/exposure of cellular components categorized as damage-associated molecular patterns (DAMPs) in the tumor immune microenvironment (TIME).^84^ Cabozantinib’s effects on the migration and phenotype of moDCs, leading to tumor suppression, support its use with ICB.^85^ Cabozantinib is currently FDA approved for use in medullary thyroid cancer, which is primarily RET driven, and has shown some clinical activity in mCRPC patients.^86^ In an ongoing phase Ib clinical trial, COSMIC-021 (NCT03170960), atezolizumab (PD-L1 inhibitor), and cabozantinib combination in mCRPC patients with metastatic progression after enzalutamide or abiraterone treatment supported the synergistic effect of this combination. A phase III trial (CONTACT-02; NCT04446117) to compare the effects of cabozantinib combination with atezolizumab in CRPC patients previously treated with AR antagonists is underway (Table 1). As previous clinical trials of atezolizumab with enzalutamide showed longer PFS in mCRPC patients with established immune patterns, including increased PD-L1 and CD8 expression,^87^ the CONTACT-02 trial is expected to provide improved clinical outcomes. The combination of cabozantinib and nivolumab (anti-PD-1) is currently being evaluated in patients with mHSPC in the CABIOS clinical trial.^88^

### PCI-32765 (ibrutinib)

Bruton’s tyrosine kinase (BTK), a non-receptor tyrosine kinase belonging to the TEC cytoplasmic tyrosine kinase family,^89^ is expressed in hematopoietic cells and is responsible for B cell growth, survival, and differentiation dynamics. During proximal BCR signaling, LYN phosphorylates CD19 (BCR co-receptor), and phospholipase Cγ2 (PLCγ2) phosphorylation by BTK leads to enhanced calcium flux and activation of multiple signaling cascades involving NF-κB and MAPK,^90^ along with alteration of chemokine receptors, including CXCR4,^91^ and integrins,^92^ in the TIME. Although BTK (BTK-A) manifests in the development of B-lineage malignancies, solid tumors, including breast cancer^93^ and PCa,^94^ have been shown to have increased expression of BTK-C, an isoform of BTK that contributes to cancer progression. The BTK inhibitor PCI-32765 (ibrutinib) is FDA approved for B cell lymphoma patients^95^ and counteracts immune suppression by myloid derived suppressor cells (MDSCs) in the tumor microenvironment.^96^ Pre-clinical evaluation of ibrutinib showed suppression of matrix metalloproteinases-2 and -9 leading to the inhibition of PCa cell migration upon treatment.^97^ A phase II trial of neoadjuvant ibrutinib in PCa patients opting for radical prostatectomy is now ongoing,^98^ and ibrutinib is now one of the first TKIs with immune modulatory activity in PCa.

### ESK981 (CEP-11981)

ESK981 is a multi-kinase inhibitor that targets VEGFR1, VEGFR2, and TEK/Tie-2 kinases and is currently in pre-clinical and early-stage clinical trial.^99^ A phase I clinical trial in advanced, relapsed, or refractory solid tumors showed promising results.^100^ Recent studies have shown that ESK981 also targets PIKfyve (FYVE-type zinc-finger-containing phosphoinositide kinase), which is involved in endosome carrier vesicle biogenesis and endomembrane homeostasis.^101^^,^^102^ In pre-clinical mCRPC models, ESK981 inhibited autophagy through PIKfyve, resulting in enhanced CXCL10 levels through the interferon-γ (IFN-γ) pathway, improving functional T cell infiltration. The immunocompetent tumor microenvironment enabled by PIKfyve inhibition synergized with ICB therapeutic response,^103^ suggesting autophagy inhibition as a treatment with significant clinical benefit. A combination study of ESK981 and nivolumab in mCRPC patients is in clinical trial (NCT04159896).

### PLX3397 (pexidartinib)

Pexidartinib is a multi-kinase inhibitor that targets CSF1R, KIT, and FLT3. Myeloid cells express CSF1R, a receptor tyrosine kinase that induces the survival, migration and differentiation of myeloid cells, including toward tumor-associated macrophages (TAMs) through the initiation of a phosphorylation cascade upon dimerization by binding of IL-34 and CSF1 ligands.^104^ These differentiated M2 macrophages are immunosuppressive and secrete cytokines, including IL-4, IL-13, TGF-β, and IL-10. Pexidartinib showed favorable inhibition of radiotherapy-induced tumor-infiltrating myeloid cells in PCa models and patients^105^; however, the outcome of a phase I clinical trial of pexidartinib combined with radiotherapy and androgen deprivation did not yield the anticipated results.^106^ Another combination study with docetaxel showed inhibition of monocyte recruitment, subsequent TAM generation, and prevention of CXCR4-CXCL12 signaling, thereby overcoming immune tolerance in CRPC and sensitizing cancer cells to docetaxel treatment.^107^

### TP-3654 (SGI-9481)

Pro-viral integration of the Moloney murine leukemia virus 1 (PIM-1) kinase is overexpressed in PCa patients.^108^ PIM-1 enhances cell-cycle progression by phosphorylating cell-cycle proteins,^109^ promoting tumor growth through c-MYC transcriptional activity,^110^ and inhibiting apoptosis.^111^ In addition, PIM-1 contributes to cancer cell invasion by phosphorylating actin-capping proteins^112^ and n-MYC downstream-regulated gene 1 (NDRG1) at serine 330, affecting its AR interaction, which directly correlates with the advanced stage of PCa.^113^ PIM-1 can also phosphorylate and alter AR transcription through its co-activator 14-3-3ζ, recruiting multiple co-regulatory proteins.^114^ PIM-1 overexpression causes immune evasion and impairs TCRβ rearrangement leading to the development of CD4^+^CD8^+^ double-positive T cells, which enables TCR checkpoint bypass, causing deregulated T cell differentiation.^115^ The first-generation PIM-1 antagonist SGI-1776 displayed cardiotoxicity (NCT00848601); however, a second-generation PIM inhibitor, TP-3654 (SGI-9481), exhibited decreased cardiotoxicity and improved potency. Successful studies demonstrating the synergy of PI3K^116^^,^^117^ or FLT3 and PIM co-targeting in PCa would favor PIM inhibitors in clinical trials.

### ACK1 inhibitor (*R*)-9b

ACK1 protein encoded by the *TNK2* gene is a ubiquitously expressed non-receptor tyrosine kinase that is overexpressed in multiple tumors, including prostate and breast, where its expression is correlated with increased invasiveness and poor prognosis.^118^^,^^119^^,^^120^^,^^121^^,^^122^^,^^123^^,^^124^^,^^125^ ACK1 is activated by multiple growth factors, including PDGF, FGF, EGF, heregulin, GAS6, and insulin, and its knockdown increases apoptosis.^121^^,^^125^ ACK1 plays a crucial role in increasing WDR5/MLL2 complex-mediated AR transcriptional activation by phosphorylating histone H4 at tyrosine 88 (pY88-H4) in the AR enhancer region.^3^ Consistent with these data, inhibition of ACK1 using the small-molecule inhibitor (*R*)-9b reversed pY88-H4 epigenetic marks at the *AR* locus, reducing AR and AR-V7 levels in mCRPC.^3^ Further mechanistic studies revealed that targeting ACK1 kinase may be a holistic therapeutic strategy to mitigate PCa due to the multi-functional role of ACK1 in tumor initiation and progression and as an effector of acetylated HOXB13, an oncogenic transcription factor associated with CRPC development.^4^^,^^5^^,^^126^ Recent studies revealed enhanced AR acetylation at Lys609 in CRPCs, which was dependent upon AR Tyr267 phosphorylation by ACK1.^5^ Further, ACK1 also phosphorylates ATP synthase F1 subunit α (ATP5F1A) in CRPC,^126^ and (*R*)-9b treatment not only significantly compromised AR transcriptional activity, but also mitigated increased mitochondrial energy output in cancer, diminishing prostate tumor growth. Interestingly, ACK1 also seems to influence immune cells; it negatively regulates T cell activation through Tyr18 phosphorylation of C-terminal Src kinase (CSK), promoting inhibitory Tyr505 phosphorylation of LCK, thereby compromising antitumor immunity.^127^ (*R*)-9b not only revitalizes T cell priming by lowering the TCR activation threshold, but also increases expression of the leukocyte attractant CXCL10. Thus, (*R*)-9b has emerged as a potent CRPC inhibitor that possesses a unique ability to overcome ICB resistance in prostate tumors.^127^

In summary, small-molecule kinase inhibitors hold significant potential to harness the power of the host immune system to fight cancers, especially in advanced metastatic therapy-resistant PCa. Prevalently, small-molecule inhibitors used for targeted cancer therapy face the major challenge of acquired drug resistance and low efficiency due to sensitivity in a limited number of patients. Nevertheless, exploration of kinase inhibitor combinations with immune therapies could improve sensitivity and efficacy, comprehensively adding to the immune-based PCa therapy mission by overcoming immune evasion and resistance during tumorigenesis.

## Circulating tumor cells

The low mutational burden and weak neoantigen expression of PCa^128^ reduces immune attraction and compromises cross-priming of tumor-infiltrating lymphocytes (TILs), leading to immune evasion by mCRPCs. Characterization of sloughed-off metastatic tumor cells termed circulating tumor cells (CTCs),^129^ provides an opportunity to improve immunotherapy owing to the potential of CTCs as real-time biomarkers of the responsive tumor microenvironment (Figure 1). CTC analysis will provide global information of multiple lesions rather than single-site biopsy. Moreover, as many trials recruit heavily pre-treated patients exhibiting altered marker status and therapy resistance, serial profiling of CTCs pre- and post-treatment for biomarkers will provide quantitative measurements of heterogeneity to apply novel drug combinations for interventional therapy.

Recently, PCa CTC profiling revealed increased B7-H3 and PD-L1 inhibitory receptor expression, compared with relatively low PD-L2 and CTLA-4 expression across disease states.^130^ Similarly, PSMA-expressing CTCs correlated inversely with PSA changes and were indicative of minimal treatment response in CRPC patients.^131^ Further, analysis of CTC heterogeneity in mCRPC improved the treatment outcome of taxane chemotherapy^132^ and ipilimumab and nivolumab immunotherapy.^133^ Molecular and digital pathology in metastatic genitourinary cancer patients treated with combination immunotherapy indicated that high CTC burden (pan-CK/CD45/PD-L1 expression) and low CD4/CD8 T cell ratios were associated with reduced survival.^134^ mCRPC patients with high microsatellite instability had profound responses to pembrolizumab.^135^ Thus, targeting CTCs could increase the vulnerability of resistant tumors to immunotherapy. Further understanding of tumor cell dissemination and CTC detection could aid the identification of proficient immune-CTC interfaces for effective therapeutic intervention.

## Caveats in PCa immunotherapy

Despite strenuous efforts to use immunotherapy as a treatment modality for PCa, desirable outcomes are yet to be achieved due to multiple limiting factors. Although CAR T therapy holds promise, the low mutational burden of PCa leads to minimal detection of novel tumor antigens as targets for therapy. In addition, low oxygen, low pH, and immunosuppressive cytokines nullify the potency of cell-based therapies, and modifications to enhance persistence and homing are warranted. Studies have shown that catalase conjugation^136^ in CAR T cells increases their persistence and immunogenicity, mitigating the immunosuppressive environment. The idea of combining oncolytic virus administration with CAR T cell therapy has been proposed to have beneficial outcomes by improving immune cell infiltration into solid tumors.^137^ This efficacy can be attributed to the lytic effect of the oncolytic viruses releasing TAA and, in turn, activating adaptive responses, including effector T cell activation, and ensuring inhibition of immune escape. However, CAR T cells alone or the concept of CAR T cell-oncolytic virus combination poses a risk of chronic immune activation^138^^,^^139^ leading to tonic signaling and cytokine release syndrome and could cause the production of autoimmune antibodies. Thus, mitigating methods must be designed and tested. Efforts to minimize off-tumor/on-target effects on normal tissue expressing the same antigen, other than the targeted tumor cells,^140^ are essential. Identification of antigens that are almost absent from normal tissue while overexpressed in cancer conditions could greatly improve clinical efficacy.

One of the main reasons for the minimal clinical efficacy of immunotherapy in PCa is overall disease heterogeneity. Precise determination of PCa constituents and its immune microenvironment could strengthen the accuracy of prognostic disease assessment, eliminate varied responses to PCa immunotherapy, and allow for concrete advances toward rational design of individualized precision medicine. Also, the PCa microenvironment is characterized by a high number of macrophages, particularly the M2 subtype, which are associated with high Gleason scores and a poor prognosis.^141^ In addition, analysis of TILs from prostate tumor samples also shows populations of T regulatory cells (Tregs) that are self-tolerant and produce marked immune inhibition, namely, classical CD4^+^ Tregs (CD4^+^CD25^+^) and less frequent CD8^+^ Tregs (CD8^+^FoxP3^+^), revealing active immune suppression mechanisms within the PCa tumor microenvironment.^142^

In addition, identification and correlation of novel immune-related markers to basic PCa phenotypes could improve current treatment strategies. Expression of immunoregulatory proteins such as B7-H3 (CD276) and HHLA2 was shown to be higher than PD-L1 expression, directly proportional to the Gleason score and tumor stage and negatively proportional to the number of CD8^+^ TILs.^143^ A 24-year follow-up study in PCa showed that the use of cholesterol-lowering statins lowers the incidence of lethal PCa in PTEN-null cancers and enriches TCR signaling genes in normal tumor-adjacent prostate tissue, opening up a rationale for studying statins as immune modulators in PCa.^144^

### Androgen as an immunosuppressant

Classical ADT to inhibit prostate tumor growth also affects tumor-associated T cells by influencing T cell-intrinsic AR signaling, preventing T cell exhaustion and overcoming immunotherapy resistance. AR inhibition in pre-clinical models sensitizes tumor-bearing mice by enhancing functional CD8^+^ T cells, increasing IFN-γ expression and augmenting response to PD-1 therapy.^145^ Moreover, a dual-phosphorylated form of sterol regulatory element-binding protein 1 pY673/951-SREBF1 has recently been shown to act as an androgen sensor that recruits KAT2A/GCN5 to deposit H2A-K130ac epigenetic marks to promote *de novo* lipogenesis and androgen synthesis. CD8^+^ T cells have robust AR expression, and tumor-derived androgen causes a paracrine effect on T cells by inhibiting nuclear translocation of SREBF1. Tumor-derived androgen also alters IFN-γ and increases the expression of the exhaustion markers PD-1 and Lag3, effects reversed upon simultaneous inhibition of KAT2A and Tyr kinases.^146^ Further exploration of T cell dynamics as a response to androgen signaling could aid in devising improved treatment plans, and thus, co-targeting of histone deacetylases and tyrosine kinases could be an effective therapeutic strategy.

### Immunotherapy of bone-metastatic prostate cancer

Bone-metastatic PCa is an incurable form of the disease, and 90% of recurrent PCa cases have bone involvement. The highly immunosuppressive bone microenvironment characterized by the presence of inflammatory monocytes, tumor-promoting M2 macrophages, enhanced exhaustion of infiltrating T cells, and increased Treg population challenges the available options for immunotherapy.^147^ Primitive knowledge of myeloid cell heterogeneity and their involvement in shaping tumor-adaptive immune scenarios complicates targeted therapy, unlike treating tumors at the primary site. A recent focus on the characterization of PCa bone metastases shows that this immune-refractory nature involves T cell exhaustion activated by the CCL20-CCR6 chemokine signaling axis and infiltration of M2 macrophages. Blockade of these signals in a pre-clinical mouse model improved survival by reinvigorating T cells.^148^ Further studies delineating the myeloid cell/T cell/tumor cell signaling nexus will offer new combinatorial possibilities to augment existing immunotherapy.

### Conclusion

Although there has been slow progress in PCa immunotherapy, consistent efforts to study the molecular features of the tumor and its surrounding environment hold promise to identify factors impeding immune intervention in PCa. Identification and testing of novel combinatorial strategies such as adjuvants and newer mechanism-based targets could overcome existing limitations, enabling long-lasting responses and improved prognosis to curb even the most difficult-to-treat PCa cases.
